# Clinical and genetic diversities of Charcot‐Marie‐Tooth disease with MFN2 mutations in a large case study

**DOI:** 10.1111/jns.12228

**Published:** 2017-07-30

**Authors:** Masahiro Ando, Akihiro Hashiguchi, Yuji Okamoto, Akiko Yoshimura, Yu Hiramatsu, Junhui Yuan, Yujiro Higuchi, Jun Mitsui, Hiroyuki Ishiura, Ayako Umemura, Koichi Maruyama, Takeshi Matsushige, Shinichi Morishita, Masanori Nakagawa, Shoji Tsuji, Hiroshi Takashima

**Affiliations:** ^1^ Department of Neurology and Geriatrics Kagoshima University Graduate School of Medical and Dental Sciences Kagoshima Japan; ^2^ Department of Neurology, Graduate School of Medicine The University of Tokyo Tokyo Japan; ^3^ Department of Pediatric Neurology Aichi Prefectural Colony Central Hospital Aichi Japan; ^4^ Department of Pediatrics Yamaguchi University Graduate School of Medicine Yamaguchi Japan; ^5^ Department of Computational Biology and Medical Sciences, Graduate School of Frontier Sciences The University of Tokyo Chiba Japan; ^6^ Department of Neurology, North Medical Center Kyoto Prefectural University of Medicine Kyoto Japan

**Keywords:** Charcot‐Marie‐Tooth disease, clinical features, Japanese, MFN2, simultaneous mutation

## Abstract

Charcot‐Marie‐Tooth disease (CMT) constitutes a heterogeneous group affecting motor and sensory neurons in the peripheral nervous system. MFN2 mutations are the most common cause of axonal CMT. We describe the clinical and mutational spectra of CMT patients harboring MFN2 mutations in Japan. We analyzed 1,334 unrelated patients with clinically suspected CMT referred by neurological and neuropediatric departments throughout Japan. We conducted mutation screening using a DNA microarray, targeted resequencing, and whole‐exome sequencing. We identified pathogenic or likely pathogenic MFN2 variants from 79 CMT patients, comprising 44 heterozygous and 1 compound heterozygous variants. A total of 15 novel variants were detected. An autosomal dominant family history was determined in 43 cases, and the remaining 36 cases were reported as sporadic with no family history. The mean onset age of CMT in these patients was 12 ± 14 (range 0–59) years. We observed neuropathic symptoms in all patients. Some had optic atrophy, vocal cord paralysis, or spasticity. We detected a compound heterozygous MFN2 mutation in a patient with a severe phenotype and the co‐occurrence of MFN2 and PMP22 mutations in a patient with an uncommon phenotype. MFN2 is the most frequent causative gene of CMT2 in Japan. We present 15 novel variants and broad clinical and mutational spectra of Japanese MFN2‐related CMT patients. Regardless of the onset age and inheritance pattern, MFN2 gene analysis should be performed. Combinations of causative genes should be considered to explain the phenotypic diversity.

## Introduction

Charcot‐Marie‐Tooth disease (CMT) is one of the most common inherited peripheral neuropathies. The prevalence of CMT was reported to be 1 per 1,215–10,300 persons (Barreto et al., 2016 ). To date, more than 80 causative genes have been reported to be associated with CMT (Timmerman et al., 2014 ). The clinical features of CMT can significantly vary among patients, even among those sharing the same mutation. Generally, CMT cases are classified into a demyelinating type [median MNCV (motor nerve conduction velocity) <38 m/s], an axonal type (median MNCV >38 m/s), and an intermediate type based on the MNCV of the median nerve. Most frequently, the demyelinating type is associated with a mutation in the gene PMP22, while a mutation in MFN2 is linked to the axonal type. MFN2 is a protein present in the mitochondrial outer membrane, which responds to the mitochondrial dynamics through a mitochondrial GTPase. The frequency of MFN2 mutations in CMT2 patients has been reported to be in the range of 17%–23% in Spanish, French, Korean, and Chinese populations (Calvo et al., 2009 ; Casasnovas et al., 2010 ; Choi et al., 2015 ; Xie et al., 2016 ). However, its frequency was reported to be lower, between 8.6% and 11%, in previous Japanese reports (Kijima et al., 2005 ; Abe et al., 2011 ).

MFN2 mutation causes typical CMT2, which is called CMT2A2, and can also present different clinical phenotypes, including hereditary motor sensory neuropathy (HMSN) with pyramidal features (HMSN V), HMSN with optic atrophy (HMSN VIA), AR‐CMT, severe early onset axonal neuropathy, early onset stroke without neuropathy, HMSN with cognitive impairment, and brain mitochondrial dysfunction (Mostacciuolo et al., 2000 ; Zuchner et al., 2006 ; Chung et al., 2008 ; Del Bo et al., 2008 ; Nicholson et al., 2008 ; Polke et al., 2011 ). In this case series, we identified novel pathogenic mutations and investigated variations in the clinical features of CMT patients due to MFN2 variants.

## Methods and Materials

### Patients

We analyzed 1,334 unrelated patients/families with clinically suspected CMT. The clinical data and DNA samples were collected from neurological and neuropediatric departments throughout Japan between 2007 and 2016. All the demyelinating patients were enrolled in this study after confirming them to be negative for PMP22 duplication/deletion as identified using fluorescence in situ hybridization and multiplex ligation probe amplification. We extracted genomic DNA using QIAGEN's Puregene Core Kit C (Qiagen, Valencia, CA, USA) or Oragene DNA self‐collection kit (DNA Genotech, Ottawa, Ontario, Canada) and carried out mutation screening tests using DNA microarray, targeted resequencing, and whole‐exome sequencing. Candidate variants detected by these methods were validated using Sanger sequencing. If available, segregation analysis was performed for those cases.

The study protocol was reviewed and approved by the institutional review board of Kagoshima University. All patients and family members provided written informed consent to participate in this study, including for the genetic analyses.

### Microarray chip sequencing

We designed a customized MyGeneChip^®^ CustomSeq^®^ Resequencing Array (Affymetrix, Inc., Santa Clara, CA, USA) to screen 30 disease‐causing genes for CMT and related diseases. We performed mutation screening using Microarray chip sequencing in patients who were enrolled from 2007 to 2012. The detailed methodology has been described elsewhere (Hashiguchi et al., 2014 ). Table S1, Supporting Information includes the sequences of 30 target genes.

### Targeted resequencing

We performed mutation screening of 60 or 72 known/candidate CMT‐related genes using two methods: the Illumina Miseq platform (Illumina Inc., San Diego, CA, USA) and the Ion Proton using a custom Ion Ampliseq^M^ panel and the Ion PI Chip kit v2 BC (ThermoFisher Scientific, Inc., Waltham, MA, USA). We performed mutation screening using Illumina Miseq platform from 2012 to 2014 and Ion Proton platform from 2014 to 2016. After aligning and mapping variant calling, we annotated and filtered variants using the CLC genomics Workbench software program (Qiagen, Hilden, Germany). We filtered out the variants with low read depth (<10) and low quality (<20). The detail methods have been previously described (Maeda et al., 2014 ; Higuchi et al., 2016 ). The symbols of the 60 and 72 target genes are shown in Table S1.

### Whole‐exome sequencing

Exome sequences were enriched using a SureSelect V4+UTRs or v5+UTRs Kit (Agilent Technologies, Santa Clara, CA, USA) and were subsequently subjected to sequencing on a hiseq2000 platform (Illumina). Sequence data was aligned to the human genome data (NCBI37/hg19), and variant calling was performed using Burrows Wheeler Aligner and SAM tools (Higuchi et al., 2016; Li and Durbin, 2009 ; Li et al., 2009 ). The called variants were annotated using the CLC genomics Workbench software program and in‐house script, and the variants with low read depth (<10) and low quality (<20) were filtered out.

### Data analysis for the determination of pathogenic mutations

We confirmed the previously reported pathogenic mutations by reference to the Human Gene Mutation Database Professional 2016.3 (https://portal.biobase-international.com/hgmd/pro). Moreover, we extracted variants that were not observed in global control databases [dbSNP (https://www.ncbi.nlm.nih.gov/SNP), 1000genome (http://browser.1000genome.org), Exome Sequencing Project (http://evs.gs.washington.edu/EVS), and Exome Aggeregation Consortium (http://exac.broadinstitute.org/)] and Japanese control database [iJGVD; integrative Japanese Genome Variation Database (https://ijgvd.megabank.tohoku.ac.jp), HGVD; Human genetic variation (http://www.hgvd.genome.med.kyoto‐u.ac.jp)] and in‐house not CMT database. Moreover, we performed in silico analysis using SIFT (http://sift.jcvi.org), POLYPHEN2 (http://genetics.bwh.harvard.edu/pph2), PROVEAN (http://provean.jcvi.org/index.php), Mutation Assessor (http://mutationassessor.org), and Condel (http://bg.upf.edu/fannsdb). We evaluated the detected variants using the American College of Medical Genetics and Genomics (ACMG) standards and guidelines (Richards et al., 2015 ).

## Results

### Epidemiology of MFN2 in Japan

**Figure 1:**
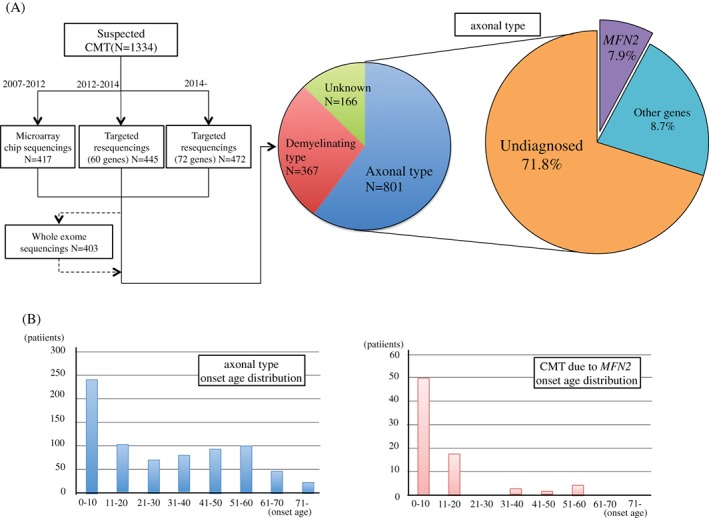
Study flow chart of this and onset age distribution. (A) Study flow chart and the rate of MFN2 mutations in axonal Charcot‐Marie‐Tooth disease (CMT). Onset age distribution for the axonal type and cases with MFN2 mutation.

We analyzed 1,334 unrelated patients with clinically suspected CMT. We detected 30 known pathogenic mutations, 1 novel pathogenic mutation, 14 novel likely pathogenic variants of MFN2, from which 44 were heterozygous mutations and 1 was compound heterozygous mutation. Of the CMT patients without PMP22 duplication/deletion, 801 patients (60%) presented the axonal phenotype, and 367 patients (28%) were classified as having the demyelinating type. We could not classify 166 patients due to a non‐recordable median compound motor action potential (CMAP) or no electrophysiological data. These CMT patients with axonal phenotype showed an MFN2 mutation rate of 8%, 63/801 (Fig. 1A).

### Clinical features

Data regarding previously reported mutations are included in Table S2. Table 1 shows the clinical data for novel pathogenic variants and likely pathogenic variants. The mutations appeared to be sporadic in 36 patients (46%) and presented an autosomal dominant inherited pattern in 43 patients (54%). The average age of onset was 12 ± 14 (range, 0–59) years and lower than that of the patients presenting the axonal phenotype [30 ± 22 (range, 0–79) years; p < 0.01]. The tendency for juvenile onset is shown in Fig. 1B. The mean onset age of sporadic cases was lower than that of autosomal dominant cases [age of onset for sporadic cases = 7.6 ± 10 years (range 0–59) years; age of onset for autosomal dominant cases = 15 ± 16 (range, 1–57); p value = 0.017].

**Table 1:**
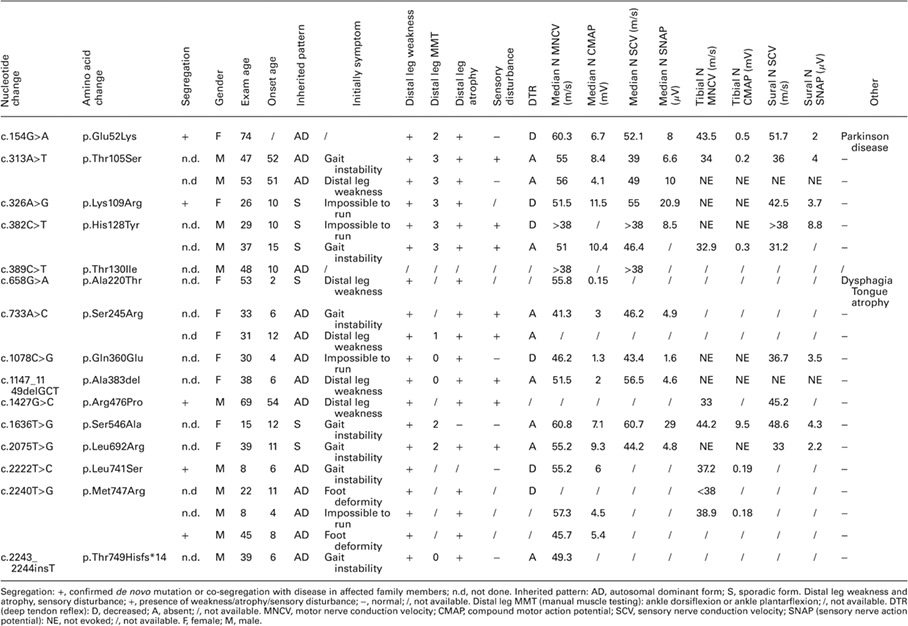
Clinical information of the cases with novel pathogenic mutations and likely pathogenic variants.

Most patients had developed motor symptoms such as distal leg weakness, foot deformity, gait instability, impossibility to run, or delayed motor milestone by the time of recruitment. Most patients presented with distal weakness, distal atrophy, and hyporeflexia [99% (75/76), 96% (72/75), and 98% (60/61), respectively]. In addition, the frequency of sensory disturbance was lower than that of any other symptoms (61% 33/54). We could evaluate sensory modality in 35 patients of these; 19 showed a decrease in sensory detection only regarding vibration, 8 showed a decrease in both vibration and superficial sensation, and 6 did not show any decrease in either of these parameters.

Only one patient with *MFN2* p.Leu710Pro mutation showed HMSN with significant spasticity and increased patellar tendon reflex, which is the HMSN V phenotype, while four patients with *MFN2* p.Arg104Trp, p.Arg104Leu, and p.Arg364Trp mutations showed HMSN with optic atrophy. Two patients with *MFN2* p.Arg364Trp mutation had vocal cord paralysis. The patient with p.Arg280His and p.Arg250Trp compound heterozygous mutations had an earlier onset than the patients with heterozygous p.Arg280His mutation.

### Electrophysiological findings

In the nerve conduction study, the MNCV of the median nerve was 51 ± 7.5 (range, 23–64) m/s and the CMAP was 4.9 ± 4.0 (range, 0–11.7) mV. In addition, the MNCV value for the tibial nerve was 38 ± 8.2 (range, 13–51) m/s and the CMAP was 0.5 ± 1.8 (range, 0–12) mV. Four patients had <38 m/s MNCV and were classified as having demyelinating type; two of them had mild MNCV reduction, 35 and 38 m/s. One patient had significantly decreased MNCV, 23 m/s. The median MNCV of four patients was unknown, and two of them had <38 m/s tibial MNCV. In nine patients, the absence of CMAP did not allow MNCV measurement.

### Genetic findings

**Figure 2:**
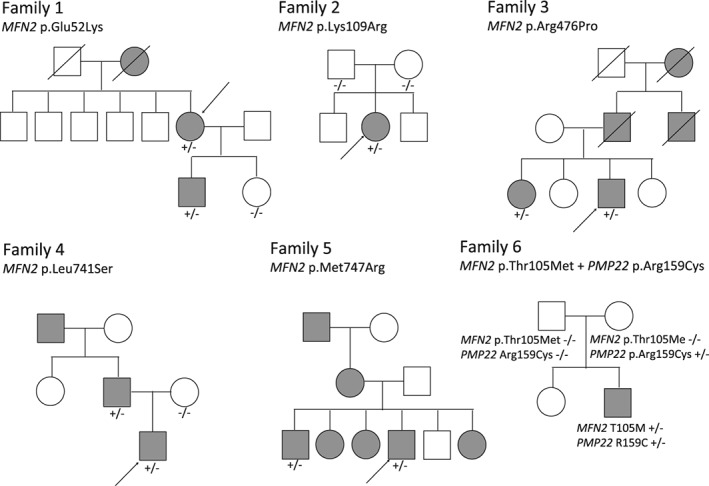
The pedigree of novel variants and simultaneously variants with segregation study. Families 1–5 indicate pedigree chart with novel variants and Family 6 indicate family with simultaneously MFN2 and PMP22 mutation. Arrow indicates probands.

We detected 1 novel pathogenic variant and 14 novel likely pathogenic variants in 20 patients (Table 2). The novel pathogenic variant was p.Lys109Arg (one patient). The novel likely pathogenic variants were p.Glu52Lys (one patient), p.Thr105Ser (two patients), p.His128Tyr (two patients), p.Thr130Ile (one patient), p.Ala220Thr (one patient), p.Ser245Arg (two patients), p.Gln360Glu (one patient), p.Ala383del (one patient), p.Arg476Pro (one patient), p.Ser546Ala (one patient), p.Leu692Arg (one patient), p.Leu741Ser (one patient), p.Met747Arg (three patients), and p.Thr749Hisfs*14 (one patient). Furthermore, we identified 30 previously reported mutations in 59 unrelated patients. Fig. 2 shows the five pedigrees of segregated novel variants as well as the family with co‐occurrence of a *de novo MFN2* mutation and a maternal *PMP22* mutation in the proband. All variants of uncertain significance are listed in Table S3.

**Table 2:** Genetic information of the novel pathogenic and likely pathogenic variants.

									ACMG			
Nucleotide change	Amino acid change	SIFT	PP2	PROVEAN	MA	Condel	Same codon reference	Reference AA change	Strong	Moderate	Support	Classification
c.154G>A	p.Glu52Lys	0.003	0.901	−2.97	2.58	0.64	No	—	—	PS4‐modarate, PM2	PP1, 3, 4	Likely pathogenic
c.313A>T	p.Thr105Ser	0	1	−3.68	3.53	0.57	*Kijima et al*. *(* 2005 *)* *Sitarz et al*. *(* 2012 *)* *Brozkova et al*. *(* 2013 *)*	Thr105Met Thr105Ala Thr105Arg	—	PS4‐modarate, PM2, 5	PP3, 4	Likely pathogenic
c.326A>G	p.Lys109Arg	0	1	−2.66	4.065	0.78	No	—	PS2	PS4‐modarate, PM2	PP3	Pathogenic
c.382C>T	p.His128Tyr	0	0.987	−5.52	3.375	0.64	*Calvo et al. (* 2009 *)*	His128Arg	—	PS4‐modarate, PM2, 5, 6	PP3	Likely pathogenic
c.389C>T	p.Thr130Ile	0	0.975	−5.42	3.94	0.68	No	—	—	PS4‐modarate, PM1, 2	PP3, 4	Likely pathogenic
c.658G>A	p.Ala220Thr	0	0.975	−3.49	2.575	0.59	No	—	—	PS4‐modarate, PM2, 6	PP3	Likely pathogenic
c.733A>C	p.Ser245Arg	0.003	0.57	−3.18	2.23	0.55	No	—	—	PS4‐modarate, PM1, 2	PP3, 4	Likely pathogenic
c.1078C>G	p.Gln360Glu	0.003	0.81	−2.44	3.07	0.62	No	—	—	PS4‐modarate, PM1, 2	PP3, 4	Likely pathogenic
c.1147_1149delGCT	p.Ala383del	—	—	−6.62	—	—	Muglia *et al. (* 2007 *)*	Ala383Val	—	PS4‐modarate, PM1, 2	PP4	Likely pathogenic
c.1427G>C	p.Arg476Pro	0.006	0.94	−2.83	2.34	0.58	No	—	—	PS4‐modarate, PM2	PP1, 3, 4	Likely pathogenic
c.1636T>G	p.Ser546Ala	0	0.999	−2.64	3.08	0.58	No	—	—	PS4‐modarate, PM2, 6	PP3	Likely pathogenic
c.2075T>G	p.Leu692Arg	0	1	−4.9	2.375	0.62	No	—	—	PS4‐modarate, PM2, 6	PP3	Likely pathogenic
c.2222T>C	p.Leu741Ser	0	1	−4.45	2.455	0.63	No	—	—	PS4‐modarate, PM1, 2	PP3, 4	Likely pathogenic
c.2240T>G	p.Met747Arg	0.53	0.001	−0.72	−0.05	0.48	*Calvo et al. (* 2009 *)*	Met747Thr	—	PS4‐modarate, PM2, 5	PP1, 4	Likely pathogenic
c.2243_4insT	p.Thr749Hisfs*14	—	—	—	—	—	No	—	—	PS4‐modarate, PM1, 2, 4	PP4	Likely pathogenic

AA, amino acid; ACMG, American College of Medical Genetics and Genomics; MA, mutation assessor; PP2, PolyPhen2.

*In silico* analysis cut off: SIFT <0.05, PP2 >0.9, PROVEAN <−2.5, MA >1.9, and Condel >0.47.

### Simultaneous mutations

The presence of simultaneously heterozygous mutations of different CMT disease‐causing genes is noteworthy. We detected the mutations in *MFN2* p.Thr105Met and *PMP22* p.Arg159Cys. The patient (a female) developed gait disturbances at the age of 1 year and later a mild mental retardation and an IQ of 69. No parental consanguinity or obvious family history was recorded (Fig. 2). Her parents had no neuropathic symptoms and normal cognitive function, but we could not evaluate the electrophysiological findings of her parents. At age 9 years, she had limited dorsiflexion of both feet joints and clubfoot. Due to a deformity in one foot and distal weakness, she could not walk unaided at age 11 years. Her nerve conduction study was normal for the median nerve and presented low CMAP for the tibial nerve. She was diagnosed with CMT2. The genetic analysis showed a p.Thr105Met mutation in *MFN2* and a p.Arg159Cys mutation in *PMP22*. Both mutations had been previously described in other patients; however, none of the parents showed the p.Thr105Met mutation in *MFN2*, being thus confirmed as a *de novo* mutation. Her asymptomatic mother presented the p.Arg159Cys mutation in *PMP22*.

## Discussion

In this Japanese case series, we detected *MFN2* mutations in 79 of 1,334 CMT patients without a *PMP22* deletion/duplication. The *MFN2* mutation accounted for 16% of the CMT2 patients in a Spanish cohort study, and 18% of CMT2 without *MPZ* and *GJB1* mutations in French patients *(Calvo et al.,* 2009 *; Casasnovas et al.,* 2010 *)*. In Asia, *MFN2* was the cause of CMT2 in 23% of Korean CMT2 patients and 18% of Chinese CMT2 patients *(Choi et al.,* 2015 *; Xie et al.,* 2016 *)*. The frequency of *MFN2* mutations is low, between 9% and 11%, as previously reported in a Japanese population study *(Kijima et al.,* 2005 *; Abe et al.,* 2011 *)*. Here, we show a frequency of the *MFN2* mutation of 8% (63/801) in Japanese CMT2 patients. A frequency of 28%–34% for sporadic or *de novo* mutations has been previously reported *(Verhoeven et al.,* 2006 *; Choi et al.,* 2015 *)*. However, sporadic cases reached a frequency of 46% in our study. This incidence rate is higher than that reported from other countries. The low frequency of *MFN2* mutations and high frequency of sporadic cases might indicate the influence of geographical and social distribution, although there is a possibility of an incomplete family history.

We showed an earlier onset of CMT due to *MFN2* mutation than of CMT2. Onset age for CMT2 often show a bimodal distribution with a peak at 0–20 years and another peak at 40–60 years. Onset in *MFN2* patients tended to be earlier than the average onset for all CMT patients considered together (Fig. 1B). Some patients showed the characteristic symptoms, for example, spasticity, optic atrophy, and vocal cord paralysis. One of our patients with *MFN2* p.Leu710Pro mutation showed spasticity, which has not been previously reported *(Verhoeven et al.,* 2006 *)*. We found a novel HMSN V phenotype for CMT2 with MFN2 p.Leu710Pro mutation. Patients with *MFN2* p.Arg364Trp showed an early onset of the severe phenotype with optic atrophy. In our study, only the patients with this specific mutation presented vocal cord paralysis, similar to that previously reported for this mutation *(Zuchner et al.,* 2006 *)*. The patient with the novel p.Arg220Thr variant showed the characteristic symptoms of dysphagia and tongue atrophy. Furthermore, we described a case of earlier‐onset CMT phenotype associated with p.Arg280His and p.Arg250Trp compound heterozygous mutation rather than a p.Arg280His heterozygous mutation. This proband was a sporadic case. The p.Arg250Trp heterozygous mutation was reported as causative compound heterozygous mutations with p.Arg400X *(Verhoeven et al.,* 2006 *)*. This p.Arg250Trp variant might be a genetic burden and make the phenotype more severe.

We presented the electrophysiological findings of CMT patients related to *MFN2* mutations. We observed a tendency toward more severe axonal neuropathies in the lower extremities, and the characteristic electrophysiological findings were demyelinating phenotype in some cases. CMT due to *MFN2* mutation was known to be mainly axonal phenotype, but some cases classified as demyelinating type or intermediate type were reported *(Kijima et al.,* 2005 *; Braathen et al.,* 2010 *)*. We need to consider the possibility that *MFN2* mutation will be classified as demyelinating type.

In addition, we describe 1 novel pathogenic variant and 14 likely pathogenic variants. The novel p.Lys109Arg variant was absent in global, Japanese, and in‐house databases (ACMG standards and guidelines; PS4‐Moderate, PM2). This variant was validated as a *de novo* variant *via* segregation analysis (PS2), and we classified novel p.Lys109Arg variants as pathogenic. The other novel variants were classified as likely pathogenic variants according to the ACMG standards and guidelines. It is difficult to judge the novel *MFN2* variants as pathogenic variants. Without previously reported same amino acid change variant or functional study, novel *MFN2* variants with a family history could not be classified as pathogenic variants. Therefore, we reported novel pathogenic and likely pathogenic variants. Eventually, we will need to clarify the level pathogenicity of these variants based on functional or population studies.

We described characteristic cases simultaneously presenting mutations of different CMT disease‐causing genes. Recent advances in genetic techniques, including next‐generation sequencing, have enabled the possibility to analyze a large number of genes from a large number of patients. These data have thus helped in identifying the combined effect of rare variants, their expression, and their contribution to disease burden, included *MFN2* and *MED25*, *MFN2* and *HSPB1*, and *MFN2* and *WNK1*. *(Gonzaga‐Jauregui et al.,* 2015 *)*. To this end, our study also shows how simultaneous heterozygous mutation may contribute to the level of clinical variability previously described. We presented simultaneous heterozygous mutations in *MFN2* p.Thr105Met and *PMP22* p.Arg159Cys. Both of these mutations had been reported before, and the mother of the proband appeared to have a mutation in *PMP22*. The reported onset age for patients with *PMP22* p.Arg159Cys mutation is 46 years *(Gess et al.,* 2011 *)*. Therefore, it is likely that onset had not happened yet for the mother of the proband, and the mutation may present incomplete penetrance. The patient with simultaneous mutations presented with an earlier onset age and mental retardation, which was a novel symptom in a patient with *MFN2* p.Thr105Met mutation. Similarly, *MFN2* and *PMP22* simultaneous mutations may result in a severe phenotype because of a double‐dose effect or genetic burden effect.

## Supporting information


**Table S1:** Target genes analyzed in the study.

**Table S2:** Clinical data of patients showing mutations previously reported.

**Table S3:** Variants of uncertain significance in this study.
