# Supplementary material for: Clinical and genetic diversities of Charcot‐Marie‐Tooth disease with MFN2 mutations in a large case study
Source: J Peripher Nerv Syst. 2017 Jul 30;22(3):191–9. doi: 10.1111/jns.12228 (PMC5697682; doi:10.1111/jns.12228)
Supplement: Supplementary file 1 — Table S1: Target genes analyzed in the study. [file JNS-22-191-s003.pdf]

Targeted 30genes of Microarray Chip sequencing.	*AARS*	*APTX*	*ARHGEF10*	*DHH*	*DNM2*	*EGR2*
	GAN	*GARS*	*GDAP1*	*GJB1*	*HSPB1*	*HSPB8*
	KARS	*LITAF*	*LMNA*	*MFN2*	*MPZ*	*MTMR2*
	NDRG1	*NEFL*	*PMP22*	*PRX*	*RAB7A*	*SBF2*
	SETX	*SH3TC2*	*SLC12A6*	*SOX10*	*TDP1*	*YARS*
	7 candidate gene					
Targeted resequencing using Miseq (60genes)	*AARS*	*APTX*	*ARHGEF10*	*DHH*	*DNM2*	*EGR2*
	FGD4	*FIG4*	*GAN*	*GARS*	*GDAP1*	*GJB1*
	HARS	*HK1*	*HOXD10*	*HSPB1*	*HSPB8*	*KARS*
	LITAF	*LMNA*	*MARS*	*MED25*	*MFN2*	*MPZ*
	MTMR2	*NDRG1*	*NEFL*	*PMP22*	*PRPS1*	*PRX*
	RAB7A	*SBF2*	*SETX*	*SH3TC2*	*SLC12A6*	*SOX10*
	TDP1	*TRPV4*	*TTR*	*YARS*	*20 candidate gene*	
Targeted resequencing using Ion Proton (72genes)	*AARS*	*APTX*	*ARHGEF10*	*BSCL2*	*DCAF8*	*DCTN1*
	DHH	*DHTKD1*	*DNM2*	*DYNC1H1*	*EGR2*	*FBLN5*
	FBXO38	*FGD4*	*FIG4*	*GALC*	*GAN*	*GARS*
	GDAP1	*GJB1*	*GJB3*	*GNB4*	*HARS*	*HK1*
	HOXD10	*HSPB1*	*HSPB3*	*HSPB8*	*IGHMBP2*	*INF2*
	KARS	*KIF1A*	*LITAF*	*LMNA*	*LRSAM1*	*MARS*
	MED25	*MFN2*	*MME*	*MPZ*	*MTMR2*	*NDRG1*
	NEFL	*PDK3*	*PLEKHG5*	*PMP22*	*PRPS1*	*PRX*
	RAB7A	*REEP1*	*SACS*	*SBF1*	*SBF2*	*SETX*
	SH3TC2	*SLC12A6*	*SLC5A7*	*SOX10*	*SURF1*	*TDP1*
	TFG	*TRIM2*	*TRPV4*	*TTR*	*YARS*	*7 candidate gene*
