# Supplementary material for: Clinical and genetic diversities of Charcot‐Marie‐Tooth disease with MFN2 mutations in a large case study
Source: J Peripher Nerv Syst. 2017 Jul 30;22(3):191–9. doi: 10.1111/jns.12228 (PMC5697682; doi:10.1111/jns.12228)
Supplement: Supplementary file 2 — Table S2: Clinical data of patients showing mutations previously reported. [file JNS-22-191-s002.pdf]

reported mutations in this study												
substitution	gender	exam age	onset age	inheritated pattern	initially symptom	distal weakness	distal atrophy	sensory disturbance	DTR	other	median MNCV	reference
R94Q	F	/	15	AD	gait instability	+	+	/	/	-	NE	Zuchner et al., 2004
	M	59	10	AD	drop foot	+	+	+	D	-	46.3	
	M	8	6	AD	gait instability	+	+	/	/	-	54	
	M	26	10	AD	gait instability	+	+	-	D	-	55.1	
	M	35	4	AD	gait instability	+	+	+	D	-	55.2	
R94W	F	9	3	S	foot deformity	+	+	+	D	-	51	Zuchner et al., 2006
	F	9	/	S	/	/	/	/	/	-	57.7	
	M	6	4	AD	gait instability	+	+	/	D	-	48.7	
	F	36	3	AD	gait instability	+	+	-	D	-	38	
	M	12	3	S	gait instability	+	+	-	/	-	44.6	
	M	6	5	AD	gait instability	+	+	+	D	-	45.2	
R94G	F	37	3	S	gait instability	+	+	-	/	-	50.4	Feely et al., 2011
R104W	M	4	2	S	gait instability	+	+	/	D	cataract	59.3	Brockmann et al., 2008
	F	6	4	S	drop foot	+	+	/	D	-	52.8	
	F	14	3	S	gait instability	+	+	+	D	optic atrophy	NE	
R104L	M	41	/	AD	distal leg weakness	+	+	+	D	optic atrophy	53.1	Sitarz et al., 2012
T105M PMP22 R159C	F	11	1	S	gait instability	+	+	+	D	MR	51.9	Kijima et al., 2005
T105M	F	5	4	AD	gait instability	+	+	+	D	-	56	
H128R	M	9	5	S	distal leg weakness	+	+	/	D	-	53.7	Calvo et al., 2009
H165R	F	67	57	AD	sensory disturbance	+	+	+	D	-	44	Chung et al., 2006
T232A	F	16	12	S	distal leg weakness	+	+	-	D	-	59.7	Sole et al., 2009
S249F	M	7	2	AD	gait instability	+	+	/	D	-	58.9	Abe et al., 2011
R259C	F	27	11	AD	muscle cramp	+	+	/	/	-	/	Sitarz et al., 2012
	M	17	16	S	distal leg weakness	+	+	-	D	-	59.5	
	M	48	15	AD	gait instability	+	+	/	/	-	54	
	M	34	/	S	/	/	/	/	/	-	64	
	M	57	11	S	impossible to run	+	+	+	D	-	58.8	
R259H	M	73	15	S	foot deformity	+	+	+	/	-	34.9	Khidiyatova et al., 2013
R274W	F	61	59	S	sensory disturbance	+	+	+	D	-	>38	Kotruchow et al., 2015
R280H	F	52	16	AD	gait instability	+	+	+	D	-	>38	Zuchner et al., 2004
	M	48	12	S	impossible to run	+	+	-	D	-	59.8	
	M	38	36	AD	sensory disturbance	+	+	+	D	-	55.3	
R280H R250W	M	7	3	S	delayed motor milestone	+	+	+	D	-	45.5	Zuchner et al., 2004 Verhoeven et al., 2006
F284Y	F	12	9	S	gait instability	+	+	+	D	-	>38	Kijima et al., 2005
K307E	M	36	10	AD	/	/	/	/	/	/	<38	Xie et al., 2016
T362M	F	1	0	S	floppy infant	+	-	/	D	corpus collasum dysgenesis	37.8	Chung et al., 2006
T362R	F	65	30	AD	gait instability	+	+	-	D	-	>38	Antoniadi et al., 2015
R364P	F	40	2	S	foot deformity	+	+	+	D	-	NE	Calvo et al., 2009
R364W	M	66	6	S	impossible to run	+	+	+	D	optic atrophy	NE	Zuchner et al., 2006
	M	6	2	S	gait instability	+	+	-	D	-	NE	
	M	64	5	S	distal leg weakness	+	+	+	/	vocal cord involvement	NE	
	M	9	3	S	foot deformity	+	+	/	D	-	NE	
	F	33	2	S	foot deformity	+	+	/	D	vocal cord involvement optic atrophy	NE	
	M	4	1	S	foot deformity	+	+	-	D	-	48.2	
	F	13	1	S	gait instability	+	+	+	D	-	NE	
	M	4	3	AD	gait instability	-	-	-	D	-	42	
M376I	F	61	20	AD	distal leg weakness	+	+	-	D	tremor	47.8	Engelfried et al., 2006
	M	68	18	AD	tremor	+	+	+	D	tremor	56.6	
	F	62	37	AD	impossible to run	+	+	+	D	-	58.6	
M376V	M	65	6	AD	impossible to run	+	+	-	D	-	56	Casanovas et al., 2010
	M	14	4	S	gait instability	+	+	/	D	MR	57.6	
S378P	F	49	44	AD	distal leg weakness	+	+	-	D	-	50	Brockmann et al., 2008
R418X	M	33	2	S	gait instability	+	+	+	D	-	55.6	Kijima et al., 2005
L644del	F	3	1	S	delayed motor milestone	+	+	/	D	post encephalopathy associated with RS virus infection	22.9	Choi et al., 2015
T706P	M	12	10	AD	gait instability	+	+	+	D	-	50.9	Mathis et al., 2014
L710P	M	4	1	AD	gait instability	+	+	/	-	spasticity	47	Verhoeven et al., 2006
	M	5	2	AD	foot deformity	+	+	/	D	-	47.6	
W740C	F	20	13	AD	distal leg weakness	+	+	-	/	-	>38	Calvo et al., 2009
c.475-1G>C	F	42	6	AD	impossible to run	+	+	+	D	-	40.9	Brockmann et al., 2008

AD; autosomal dominant, S; sporadic, MR; mental retardation, NE; not evoked

Abe A, Numakura C, Kijima K, Hayashi M, Hashimoto T, Hayasaka K (2011). Molecular diagnosis and clinical onset of Charcot-Marie-Tooth disease in Japan. J Hum Genet 56:364–368.

Antoniadi T, Buxton C, Dennis G, Forrester N, Smith D, Lunt P, Burton-Jones S (2015). Application of targeted multi-gene panel testing for the diagnosis of inherited peripheral neuropathy provides a high diagnostic yield with unexpected phenotype-genotype variability. BMC Med genet 16:84.

Brockmann K, Dreha-Kulaczewski S, Dechent P, Bonnemann C, Helms G, Kyllerman M, Bruck W, Frahm J, Huehne K, Gartner J, Rautenstrauss B (2008). Cerebral involvement in axonal Charcot-Marie-Tooth neuropathy caused by mitofusin2 mutations. J Neurol 255:1049-1058.

Calvo J, Funalot B, Ouvrier RA, Lazaro L, Toutain A, De Mas P, Bouche P, Gilbert-Dussardier B, Arne-Bes MC, Carriere JP, Journel H, Minot-Myhie MC, Guillou C, Ghorab K, Magy L, Sturtz F, Vallat JM, Magdelaine C (2009). Genotype-phenotype correlations in Charcot-Marie-Tooth disease type 2 caused by mitofusin 2 mutations. Arch Neurol 66:1511-1516.

Casasnovas C, Banchs I, Cassereau J, Gueguen N, Chevrollier A, Martinez-Matos J A, Bonneau D, Volpini V (2010). Phenotypic spectrum of MFN2 mutations in the Spanish population. J Med Genet 47:249-256.

Choi BO, Nakhro K, Park HJ, Hyun YS, Lee JH, Kanwal S, Jung SC, Chung KW (2015). A cohort study of MFN2 mutations and phenotypic spectrums in Charcot-Marie-Tooth disease 2A patients. Clin Genet 87:594-598.

Chung KW, Kim SB, Park KD, Choi KG, Lee JH, Eun HW, Suh JS, Hwang JH, Kim WK, Seo BC, Kim SH, Son IH, Kim SM, Sunwoo IN, Choi BO (2006). Early onset severe and late-onset mild Charcot-Marie-Tooth disease with mitofusin 2 (MFN2) mutations. Brain 129:2103-2118.

Engelfried K, Vorgerd M, Hagedorn M, Haas G, Gilles J, Epplen JT, Meins M (2006). Charcot-Marie-Tooth neuropathy type 2A: novel mutations in the mitofusin 2 gene (MFN2). BMC Med Genet 7:53.

Feely SM, Laura M, Siskind CE, Sottile S, Davis M, Gibbons VS, Reilly MM, Shy ME (2011). MFN2 mutations cause severe phenotypes in most patients with CMT2A. Neurology 76:1690 – 1696.

Khidiyatova IM, Skachkova IA, Saifullina EV, Magzhanov RV, Schagina OA, Zinchenko RA, Petrin AN, Khusnutdinova EK (2013). MFN2 gene analysis in patients with hereditary motor and sensory neuropathy from Bashkortostan Republic. Genetika 49:884-890.

Kijima K, Numakura C, Izumino H, Umetsu K, Nezu A, Shiiki T, Ogawa M, Ishizaki Y, Kitamura T, Shozawa Y, Hayasaka K (2005). Mitochondrial GTPase mitofusin 2 mutation in Charcot-Marie-Tooth neuropathy type 2A. Hum Genet 116:23–27.

Kotruchow K, Kabzinska D, Kochanski A (2015). Pathogenic mutations and sequence variants within mitofusin 2 gene in polish patients with different hereditary motor-sensory neuropathies. Acta Neurobiol Exp 75:264–278.

Mathis S, Funalot B, Boyer O, Lacroix C, Marcorelles P, Magy L, Richard L, Antignac C, Vallat JM (2014). Neuropathologic characterization of INF2-related Charcot-Marie-Tooth disease: evidence for a Schwann cell actinopathy. J Neuropathol Exp Neurol 73:223–233.

Sitarz KS, Yu-Wai-Man P, Pyle A, Stewart JD, Rautenstrauss B, Seeman P, Reilly MM, Horvath R, Chinnery PF (2012). MFN2 mutations cause compensatory mitochondrial DNA proliferation. Brain 135 e219, 1-3

Sole G, Ferrer X, Vital C, Martin-Negrier ML, Vital A, Latour P (2009). Ultrastructural mitochondrial modifications characteristic of mitofusin 2 mutations (CMT2A). J Peripher Nerv Syst14:206 – 207.

Verhoeven K, Claeys KG, Zuchner S, Schroder JM, Weis J, Ceuterick C, Jordanova A, Nelis E, De Vriendt E, Van Hul M, Seeman P, Mazanec R, Saifi GM, Szigeti K, Mancias P, Butler IJ, Kochanski A, Ryniewicz B, De Bleecker J, Van den Bergh P, Verellen C, Van Coster R, Goemans N, Auer-Grumbach M, Robberecht W, Milic Rasic V, Nevo Y, Tournev I, Guergueltcheva V, Roelens F, Vieregge P, Vinci P, Moreno MT, Christen HJ, Shy ME, Lupski JR, Vance JM, De Jonghe P, Timmerman V (2006). MFN2 mutation distribution and genotype/phenotype correlation in Charcot-Marie-Tooth type 2. Brain 129:2093–2102.

Xie Y, Li X, Liu L, Hu Z, Huang S, Zhan Y, Zi X, Xia K, Tang B, Zhang R (2016). MFN2-related genetic and clinical features in a cohort of Chinese CMT2 patients. J Peripher Nerv Syst 21:38-44.

Zuchner S, Mersiyanova IV, Muglia M, Bissar-Tadmouri N, Rochelle J, Dadali EL, Zappia M, Nelis E, Patitucci A, Senderek J, Parman Y, Evgrafov O, Jonghe PD, Takahashi Y, Tsuji S, Pericak-Vance MA, Quattrone A, Battaloglu E, Polyakov AV, Timmerman V, Schroder JM, Vance JM (2004). Mutations in the mitochondrial GTPase mitofusin 2 cause Charcot-Marie-Tooth neuropathy type 2A. Nat Genet 36:449-451

Zuchner S, De Jonghe P, Jordanova A, Claeys KG, Guergueltcheva V, Cherninkova S, Hamilton SR, Van Stavern G, Krajewski KM, Stajich J, Tournev I, Verhoeven K, Langerhorst CT, de Visser M, Baas F, Bird T, Timmerman V, Shy M, Vance JM (2006). Axonal neuropathy with optic atrophy is caused by mutations in mitofusin 2. Ann Neurol 59:276-281.
