# Supplementary material for: Clinical and genetic diversities of Charcot‐Marie‐Tooth disease with MFN2 mutations in a large case study
Source: J Peripher Nerv Syst. 2017 Jul 30;22(3):191–9. doi: 10.1111/jns.12228 (PMC5697682; doi:10.1111/jns.12228)
Supplement: Supplementary file 3 — Table S3: Variants of uncertain significance in this study. [file JNS-22-191-s001.pdf]

Nucleotide change	Amino acid change	SIFT	PROVEAN	PP2	MA	Condel	Control DB	ACMG Pathogenic	ACMG Benign
51T>G	Asn17Lys	0.106	-0.85	0	0.345	0.559894227	-	PS4-Moderate PM2, PP4	BP4
902T>C	Ile301Thr	0.001	-3.97	0.756	2.24	0.586068658	1/120296 (ExAC)	PS4-Modearte PP3, PP4	BS2
1758G>A	Met586Ile	0.538	-0.73	0.005	1.245	0.55306655	1/4028 (iJGVD)	PS4-Moderate	BS2, BP4
2179C>G	Leu727Val	0.665	0.16	0.123	-0.625	0.455770068	1/428 (HGVD)	PS4-Moderate PM6	BS2, BP4
